# Correction Notice: Isolation of Healthy F4/80+ Macrophages from Embryonic day E13.5 Mouse Fetal Liver Using Magnetic Nanoparticles for Single Cell Sequencing

**DOI:** 10.21769/BioProtoc.4329

**Published:** 2022-02-05

**Authors:** Kaustav Mukherjee, Lezanne Ooi

**Affiliations:** Department of Cell Developmental and Regenerative Biology, Icahn School of Medicine at Mount Sinai, New York, NY, USA; Black Family Stem Cell Institute, Icahn School of Medicine at Mount Sinai, New York, NY, USA; Mindich Child Health and Development Institute, Icahn School of Medicine at Mount Sinai, New York, NY, USA; Tisch Cancer Institute, Icahn School of Medicine at Mount Sinai, New York, NY, USA

After publication of https://bio-protocol.org/e4243, we noticed a mistake in this protocol which needs to be rectified -- Under "Materials and Reagents" Point 9: FWV ICAM4/αv inhibitor peptide (custom sequence LRQGKTLRG). This FWV ICAM4/αv peptide sequence is not correct, it should be **SVPFWVRMS**.

## 
References

1. Mukherjee, K. and Bieker, J. J. (2021). Isolation of Healthy F4/80+ Macrophages from Embryonic day E13.5 Mouse Fetal Liver Using Magnetic Nanoparticles for Single Cell Sequencing.
*Bio-protocol* 11(23): e4243

